# Geoaccumulation Index and Enrichment Factor of Arsenic in Surface Sediment of Bukit Merah Reservoir, Malaysia

**DOI:** 10.21315/tlsr2020.31.3.8

**Published:** 2020-10-15

**Authors:** Mohd Ilman Che Abdullah, Amir Shah Ruddin Md Sah, Hazzeman Haris

**Affiliations:** School of Biological Sciences, Universiti Sains Malaysia, 11800 USM Pulau Pinang, Malaysia

**Keywords:** Sediment, Heavy Metal, Arsenic, Geoaccumulation Index, Enrichment Factor, Mendapan, Logam Berat, Arsenik, Indeks Pengumpulan Geo, Faktor Pengayaan

## Abstract

An investigation study was conducted in Bukit Merah Reservoir (BMR) for the assessment of arsenic concentration in the surface sediment in 23 sampling stations. The sediment samples were digested and analysed for arsenic using Inductively Coupled Plasma-Optical Emission Spectrometry (ICP-OES). Sediment parameters such as pH (4.42 ± 0.71), redox potential (121.77 ± 42.45 mV), conductivity (205.7 ± 64.07 μS cm^−1^) and organic matter (25.35 ± 9.34%) were also examined. The main objectives of this study are to determine the arsenic distribution and concentration and at the same time to assess the enrichment of arsenic using the geoaccumulation index (*I_geo_*) and enrichment factor (EF). This study shows the total arsenic concentration in the surface sediment of BMR is 4.302 ± 2.43 mg kg^−1^ and found to be below the threshold value of Canadian Interim Sediment Quality Guidelines (ISQG). High arsenic concentration is recorded near the southern part of the lake where anthropogenic activities are prevalent. Based on *I_geo_*, 13% of sampling stations are categorised as moderately polluted, 52.2% as unpolluted to moderately polluted and the rest is categorised as unpolluted. EF shows 78.3% stations are classified as extremely high enrichment and the rest as very high enrichment. This finding provides important information on the status of arsenic contamination in BMR and creating awareness concerning the conservation and management of the reservoir in the future.

## Abstract (ms)

Satu kajian penyelidikan telah dilakukan di Takungan Bukit Merah (BMR) untuk menilai kepekatan arsenik pada permukaan mendapan di 23 stesen pensampelan. Sampel mendapan dicerna dan dianalisa menggunakan *Inductively Coupled Plasma-Optical Emission Spectrometry* (ICP-OES). Parameter mendapan seperti pH (4.42 ± 0.71), keupayaan redoks (121.77 ± 42.45 mV), kekonduksian (205.7 ± 64.07 μS cm^−1^) dan jirim organik (25.35 ± 9.34%) juga diuji. Objektif utama kajian ini adalah untuk mengenal pasti taburan dan kepekatan arsenik dan pada masa yang sama menilai pengayaan arsenik menggunakan indeks pengumpulan geo (*I_geo_*) dan faktor pengayaan. Kajian ini menunjukkan jumlah kepekatan arsenik pada permukaan mendapan di BMR ialah 4.302 ± 2.43 mg kg^−1^ dan didapati di bawah nilai ambang Garis Panduan Kualiti Interim Mendapan Kanada. Kepekatan arsenik yang tinggi direkodkan berhampiran selatan tasik di mana aktiviti antropogen tersebar. Berdasarkan *I_geo_*, 13% daripada stesen pensampelan dikategorikan sebagai sederhana tercemar, 52.2% tidak tercemar kepada sederhana tercemar dan bakinya dikategorikan sebagai tidak tercemar. Faktor pengayaan menunjukkan 78.3% stesen diklasifikasikan sebagai pengayaan yang sangat tinggi dan bakinya agak tinggi. Dapatan ini memberikan maklumat yang penting berkenaan status pencemaran arsenik di BMR dan mewujudkan kesedaran terhadap pemuliharaan dan pengurusan takungan ini di masa hadapan.

**Highlights**

- The total arsenic concentration in the surface sediment of Bukit Merah Reservoir is 4.302 ± 2.43 mg kg^−1^ and found to be below the threshold value of Canadian Interim Sediment Quality Guidelines (ISQG).
- Based on *I_geo_*, 13% of sampling stations are categorised as moderately polluted, 52.2% as unpolluted to moderately polluted and the rest is categorised as unpolluted.
- The enrichment factor (EF) shows 78.3% stations are classified as extremely high enrichment and the rest as very high enrichment.

## INTRODUCTION

Arsenic (As) is a highly toxic and carcinogenic metalloid that posed a serious threat to living organisms including humans. This metalloid is responsible for many accidental, occupational and therapeutic poisonings since its first discovery in 1250 (Mudhoo *et al*. 2011). Arsenic naturally distributed in the earth’s crust at an average concentration of 1.5–5.5 mg kg^−1^ (Bosch *et al*. 2016; Sakan *et al*. 2012; Shtangeeva 2005). Arsenic is ubiquitous in soil, sediment and groundwater. In unpolluted soil, the average concentration of arsenic is between 1–40 mg kg^−1^ and might reach up to 14000–27000 mg kg^−1^ in the heavily polluted soil. The average concentration of arsenic in surface sediment usually below 10 mg kg^−1^ (Huang *et al*. 2016; Loska *et al*. 2003; Sakan *et al*. 2012).

In the 20th century, the most devastating arsenic poisoning was reported in Bangladesh with 70–80 million people are affected due to groundwater contaminated with arsenic. The level of arsenic in tube-well water in the district boarding the West Bengal is recorded ranging from 150 ppb–200 ppb, nearly four times higher than permissible limit (Alam *et al*. 2002; Hassan *et al*. 2011; Riaz Uddin & Naz Hasan 2011). Arsenic can cause an acute and chronic effect in humans such as neurotoxicity, skin problems, cardiovascular disease, hematological, respiratory symptoms, developmental effects and various types of cancer (Bosch *et al*. 2016; Kapaj *et al*. 2006; Rasheed *et al*. 2016; Sanyal *et al*. 2017; Yang *et al*. 2016).

The source of arsenic might came from natural factors such as geological weathering, biological and anthropogenic. Geological weathering is the primary factor of groundwater contamination by arsenic. A high level of arsenic in groundwater can enter the food chain by the accumulation of arsenic by aquatic plant and phytoplankton and then continue to the next trophic level. Consuming of aquatic organism contaminated by arsenic such as fish is considered one of the leading factors of arsenic toxicity. Nevertheless, the source of arsenic from human activities (i.e., gold mining, smelting activities, production of semiconductor (gallium arsenide), manufacturing of arsenic-based pesticides and wood preservative) is the primary issue of arsenic pollution (Ali *et al*. 2016; Bosch *et al*. 2016; Rasheed *et al*. 2016; Roy 2010). The total amount of arsenic production through anthropogenic activities had reached 140,0000 t per year, compared to 3,000 t per year by volcanic activity, and 45,000 t per year by the natural process of rocks and soil weathering (Shtangeeva 2005).

The solubility of arsenic is influenced by soil pH, organic matter, soil mineralogy and arsenic oxidation state (Cagnin *et al*. 2017; Hooda 2010; Wang *et al*. 2016). In the natural environment, arsenic can exist in many different forms whether it is organic; methylarsonic acid (MMA^5+^) acid, methylarsonous acid (MMA^3+^) or inorganic; arsenate (As(V)), arsenite (As(III)) (Rasheed *et al*. 2016). However, in sediment the predominantly arsenic speciation in oxidising conditions is As(V) while As(III) in reducing condition. Inorganic arsenic is also relatively mobile in the soil especially in alkaline soils (Hooda 2010; Wang *et al*. 2016). As(V) is readily sorbs to the mineral in the sediment, thus less mobile and less toxic compared to As(III). Nevertheless, both inorganic arsenic is carcinogenic, mutagenic and teratogenic (Hatje *et al*. 2010). This type of inorganic arsenic is the most dangerous type to the aquatic organism and human being due to its stability and readily absorbed by gills, liver, gastrointestinal tract, abdominal cavity and muscle (Bosch *et al*. 2016; Rasheed *et al*. 2016). There is also enough evidence to associate the ingestion of inorganic arsenic in the human will lead to bladder and lung cancer (Hassan *et al*. 2011; Kapaj *et al*. 2006). Due to the hazardous effect and its widespread usage in agriculture and industrial, arsenic is classified as the number one toxin on the US Environmental Agency’s list of pollutants (Hatje *et al*. 2010).

Bukit Merah Reservoir (BMR) is the oldest man-made reservoir in Peninsular Malaysia, which function as a source of agricultural irrigation and domestic water supply. This reservoir provides water for a double cropping system of paddy fields under the Kerian Irrigation Scheme, which covers 24 000 hectares of paddy fields. This is the largest granary operated and maintained by the Malaysian Drainage and Irrigation Department. Currently, BMR also supplies freshwater for domestic and commercial demand in both Kerian and Larut Matang District with an estimation of 200,000 residents. BMR also provides a source of the inland fishery for the 50 registered fisherman with annual fish production estimated at 38 kg ha^−1^ yr^−1^ (Ambak & Jalal 2006; Hidzrami 2010). This lake also considered as the original sanctuary of the endangered golden Arowana (*Scleropages formosus*) in this country. Along with its long history, this reservoir had undergone rapid development around the perimeters of the lakes such as agriculture, tourism, sand mining and logging. For instance, palm oil and rubber plantation constitute 48.3 km^2^ and 98.9 km^2^ which represent 12% and 24% of the land used around the BMR (Hidzrami 2010). Therefore, there is a possible input of arsenic into the lake due to these anthropogenic activities. Due to these anthropogenic activities, eutrophication is the major problem faced by the reservoir. Being a lentic and eutrophic water body, this arsenic likely to accumulate and concentrate at the surface sediment of the lake and there is always a possible release of arsenic into the water and later bioaccumulate in an aquatic organism such as fish (Qin *et al*. 2016).

Although there have been a number of studies on the contamination of heavy metals in BMR in the past few years, these studies were limited to the water column, and none of these studies reported the presence of arsenic in BMR (Akinbile *et al*. 2013; Shuhaimi-Othman *et al*. 2010). The arsenic from the anthropogenic activities ultimately will enter the aquatic ecosystem in solution form. Gradually, these potentially toxic elements will bind or adsorption to particulate matter in the water column (i.e., suspended sediment, organic and inorganic colloidal particles). This particulate matter will eventually settle and accumulate into the surface sediment of the aquatic environment (Adel Mashaan *et al*. 2011; Khodami *et al*. 2017; Shafie *et al*. 2013). Therefore, assessment of sediment is crucial because it is known to act as both source of water pollution and sink for arsenic and can determine the fate, effect and transport of arsenic due to change of physico-chemical parameters (Sakan *et al*. 2012; Wang *et al*. 2016). Hence, this paper is aimed at determining the concentration and distribution of arsenic in the surface sediment of BMR.

Geo-accumulation index (*I_geo_*) and enrichment factor (EF) are widely used for the assessment of the degree of pollution and health status of the sediment. The advantage of the two indices is that they can identify whether the level of arsenic in sediment is due to anthropogenic input or natural input or a combination of both. The information provided through this study will facilitate a better understanding of the distribution and enrichment of arsenic in this area, thus provide useful preliminary information for further environmental conservation and sustainable management of other man-made lakes in this country.

## MATERIALS AND METHODS

**Figure 1:**
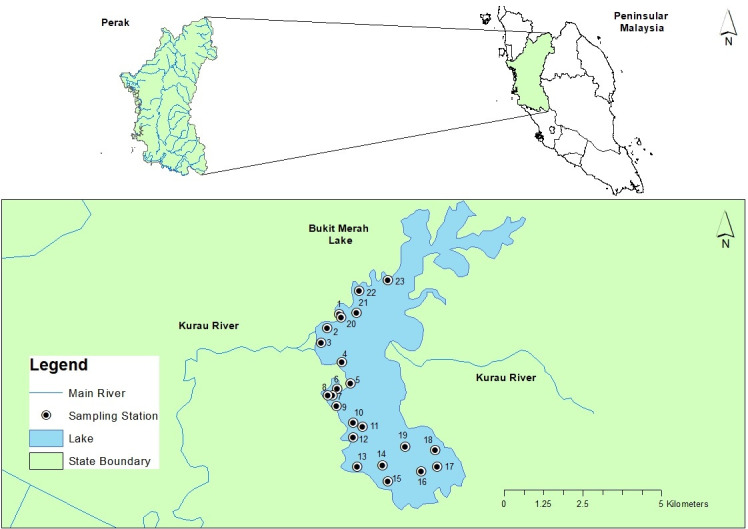
Sampling sites in BMR, Perak (Source: Google Map).

BMR is created by modified homogenous embankment method constructed in the upper stream of Kurau River and Merah River confluence in 1906 (Fig. 1). The initial height of the dam after the construction completed is 8.08 m, and then during the Second Malaysia Plan (1961 to 1965) its embankment was raised to 10.67, and the latest modification was completed in 1984, with the final height of the dam is 11.28 m. The reservoir has two spillways located on south and north where maximum discharge is estimated at 141.58–424.75 m^3^ s^−1^ respectively (Hidzrami 2010; Siti Hidayah *et al*. 2012).

BMR with surface area around 40 km^2^ is located in the district of Kerian in the Northen Perak State at a longitude of 5° 2’ 00” and latitude 100° 40’ 00” and divided into north and south lake by a 4.7 km railway. The main water source for the BMR came from Kurau River and Merah River, where Kurau River system created the largest catchment area (323.0 km^2^) and the highest elevation of about 861 m above the sea level. This is followed by the Jelutong River (7.1 km^2^) and Merah River (4.25 km^2^) and Selarong River (3.1 km^2^) (Ismail & Najib 2011).

A total of 23 sampling stations were set up, which covers both the South and North of BMR. However, certain areas on the north side of the lake were not accessible due to aquatic vegetation. All sampling was conducted during the inter-monsoon season in April 2018. A total of 23 homogenised triplicates sediment samples were collected from these sampling stations. The choice of sampling station was made based on several criteria such as it should represent the general condition of the lake section. At the same time, it also should indicate the possible anthropogenic activities around the BMR, such as plantation, residential area, tourism hot spot, fisherman jetty and source of water into the BMR from Kurau River and Merah River.

The upper layer of sediment (0 cm–5 cm) was taken using an Eakman grab in triplicates and transferred into a plastic container to be homogenised and later stored in double layers plastic bag. The sediments were temporarily stored in the icebox at 4°C before transferred to the laboratory. In the laboratory, the sediment was transferred into a separate plastic tray and left to dry at room temperature until constant weight.

In the laboratory, determination of sediment parameters such as pH, redox potential, salinity, and electroconductivity was conducted by mixing the sediment with deionised water in ratio 1:10. The slurry mixture was placed on a magnetic stirrer for 30 min and left to stand for an hour before the measurement of the parameter was taken using Eutech Cyberscan Multiparameters CD 650 and PCD 650. All of these sediment parameters were taken in triplicates. Before sampling, all the measurement devices were calibrated according to the manufacturer manual. Total organic matter (TOM) of sediment samples were determined using a lost on ignition (LOI) technique. By calculating the difference between the dry weight of the sediment sample pre and post ashing in a muffle furnace at 550°C for 4 h (Radojevic & Bashkin 2006).

For analyses of arsenic, the sediment samples were crushed into a fine powder using a porcelain mortar and pestle, which pre-washed with 10% Nitric acid (HNO_3_). The digestion method suggested by Radojevic and Bashkin (2006) and EPA Method 3050a (EPA 2007) was used in this study. The aqua regia solution was prepared by mixing 150 mL of HCI solution (130 mL concentrated HCI + 120 mL of mili-Q water) with 50 mL of concentrated HNO_3_. The samples were placed in the digestion tube and immersed overnight in 5 mL of aqua regia solution. Then the samples were digested in duplicate using a microwave digester. The temperature is set at 180°C for 9.5 min and allowed to cool at room temperature in the microwave. The digested samples were filtered with a 0.45 μm Whatman filter paper into a 50 mL volumetric flask. The total of 0.25 M HNO_3_ is added up the mark before analysed using Perkin Elmer Optima 5300 ICP-OES for the presence of arsenic. The precision of the analyses was measured using certified reference material CRM016 for freshwater sediment and the percentages of recovery ranged from 94.83%–106.38%.

The geoaccumulation index *(I_geo_*) was developed by Muller (1969) to assess the level of heavy metal and metalloid elements in the sediment by comparing the status of the current concentration with the pre-industrial level. Since then, this index has been successfully used by many researchers worldwide to evaluate the status of sediment as it easy to calculate, and the result can be straightforwardly interpreted by the public and regulation bodies (Alves *et al*. 2018; Islam *et al*. 2018; Rajeshkumar *et al*. 2018; Shafie *et al*. 2013). The index is calculated based on the equation:

$$I_{geo}={\text{Log}}_{2}\;[Ci/(1.5\;Cri)]$$

where:

*Ci* is the concentration of metals in sediment (mg kg^−1^);
*Cri* is a pre-industrial geochemical concentration or reference value of the heavy metal in particular area;

Factor 1.5 is used to reflect the possible fluctuation of the element in the background value as well as minimal anthropogenic influences or input. Based on the *I_geo_* indices, it can be classified into seven classes as shown in Table 1.

Enrichment factor (EF) is another useful index for the assessment of the enrichment level of metals and metalloid in sediment. Initially, this index was developed to assess the origin of elements in the atmosphere, seawater, and precipitation. Nowadays, this index has been successfully applied for the study of soils, marine and freshwater sediments (Goher *et al*. 2014; Shafie *et al*. 2013; Varol 2011). This universal index is a relatively simple and easy way to evaluate the enrichment degree and allow comparison of contamination of different environmental media (Nowrouzi & Pourkhabbaz 2014). At the same time, this index can be used to confirm whether heavy metals and metalloids in sediment are due to anthropogenic activities (Jahan & Strezov 2018). Enrichment factor can be classified into five categories as shown in Table 2. Calculation of enrichment factor was conducted according to the equation:

$$\text{EF}=({\text{C}}_{\text{n}}/{\text{C}}_{\text{ref}})/({\text{B}}_{\text{n}}/{\text{B}}_{\text{ref}})$$

where:

C_n_ = concentration of a measured element on the study site (mg kg^−1^);
C_ref_ = concentration of the measuring element in the reference environment. Can be based on the earth’s crust, the country average, etc. (mg kg^−1^);
B_n_ = concentration of reference element of the study site (mg kg^−1^);
B_ref_ = concentration of the reference element in the reference environment (mg kg^−1^).

Commonly used elements for normalisation are Fe, Sn, Mn or Al (Habib *et al*. 2018; Jahan & Strezov 2018; Shafie *et al*. 2013; Zahra *et al*. 2014). The normalisation is important to differentiate the trace element source that originates from anthropogenic activities and natural means. In this study, the earth’ crust concentration of ferum which is 50000 mg kg^−1^ was used as the element of normalisation for the calculation of geo-accumulation index and enrichment factor (Haris & Aris 2013). This is because nonexistence comparable baseline research has been conducted in the study area. Fe was used as the element of normalisation due to several reasons: (i) it is uniformly distributed in the natural environment and the fourth major element in the earth’s crust; (ii) commonly associated with fine solid surfaces; (iii) it is geochemistry is alike to those many trace elements (Kadhum *et al*. 2015; Varol 2011).

Normally, if the value of enrichment factor is close to or < 1, this reflects that the primary source of trace elements is from a natural source such as crustal or marine environments. However, if the enrichment factor is larger than 1, this shows that the main source is from anthropogenic activities (Adebola *et al*. 2018; Habib *et al*. 2018; Jahan & Strezov 2018).

## RESULTS

Table 3 summarised the descriptive statistic for sediment and water physico-parameters collected in BMR during the sampling period. The value of the mean was determined with standard deviation (SD) in Table 3. All parameters are measured in triplicate except for arsenic and ferum concentration (duplicate).

The mean water pH in BMR is recorded as slightly acidic (6.47 ± 0.60) and ranged from slightly alkaline at S19 (pH 7.58 ± 0.21) to acidic at S23 (pH 5.47 ± 0.23) with a coefficient of variation (CV) of 9.27 which indicates the low variability of water pH in BMR. S5 recorded the highest conductivity (29.29 ± 1.35 μS cm^−1^), TDS (24.34 ± 1.15 ppt). Meanwhile, the lowest conductivity (17.31 ± 0.51 μS cm^−1^) and TDS (15.18 ± 0.51 ppt) are recorded at S1. Analysis of sediment parameters shows that the mean pH of sediment is recorded as acidic (4.46 ± 0.77). S3 has the maximum pH (7.34 ± 0.38) and at the same time recorded the lowest redox potential (–47.87 ± 5.92 mV). The lowest pH is recorded at S19 (3.52 ± 0.0). S19 also has the highest redox potential (173.53 ± 0.06 mV), conductivity (727.73 ± 1.19 μS cm^−1^). The highest organic matter was found at S18 (46.15%) while the lowest is at S4 (16.3%). Fe was highest at S16 (3162.95 ± 61.43 mg kg^−1^) and lowest at S22 (963.21 ± 97.98 mg kg^−1^).

**Figure 2:**
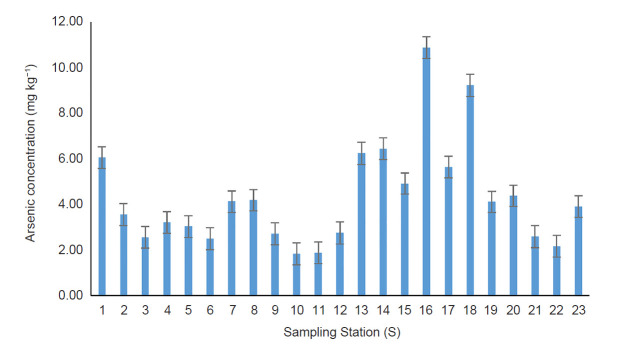
Concentration of arsenic in a sampling station in BMR. Threshold limit according to ISQGs is 5.9 mg kg^−1^.

The descriptive statistic shows that the mean of total arsenic concentration in BMR was 4.302 ± 2.43 mg kg^−1^ with a CV of 56.49%. The high value of CV indicates a high variation concentration between the sampling stations. This is comprehensible by an apparent disparity in the total arsenic concentration between the highest and lowest, which was 10.88 ± 1.81 mg kg^−1^ at S16 and 1.83 ± 1.0805 mg kg^−1^ at S10. The second highest concentration of arsenic was recorded at S18 (9.22 ± 1.70 mg kg^−1^). In general, the mean As concentration in this study (4.302 ± 2.43 mg kg^−1^) was below the ISQGs (5.9 mg kg^−1^). However, three stations (i.e., S1, S16 and S18) were found to have exceeded the guideline. Kruskal-Wallis H test on the sediment samples reveals that the total arsenic concentration among sampling stations was significantly different (*X*^2^ (22) = 36.772, *P* < 0.05). The present finding revealed a trend where most of the highest arsenic concentrations were distributed in the southern part of the lake especially at S13, S14, S16, S17 and S18 (Fig. 2).

**Figure 3:**
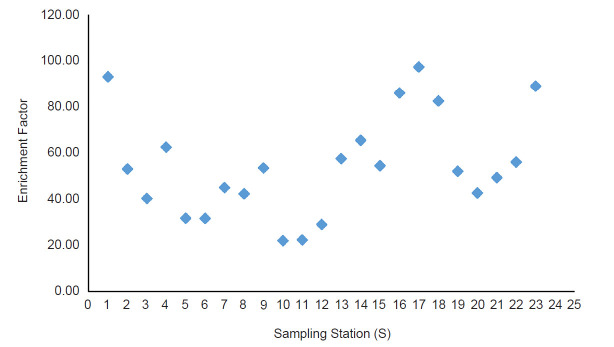
Enrichment factor of arsenic in the sediment of BMR.

**Figure 4:**
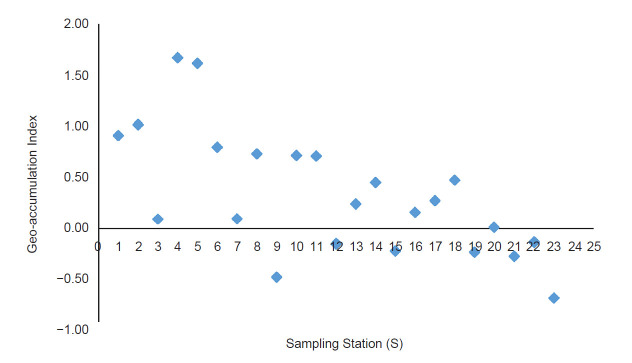
Geoaccumulation index of arsenic in the sediment of BMR.

This area is marked with dead trees, highly infested with submerged vegetation (*Capomba* sp.) and black colored sediment due to the high presence of organic matter. In the northern part of the lake, only one station (S1) recorded a high concentration of arsenic. Spearman rank analysis shows a positive correlation between the level of organic matter with the concentration of arsenic (*r_s_* (46) = 0.692, *p* < 0.05). However same analysis shows negative correlation with pH value (*r_s_* (46) = −0.367, *p* < 0.05). The values of *I_geo_* and EF for each sampling station are shown in Figs. 3 and 4. The mean values of *I_geo_* and EF were recorded at 0.28 ± 0.65 and 54.74 ± 22.23, respectively.

A comparison between the two indices shows a high disparity where the value of EF displays a higher classification of arsenic enrichment in BMR sediment compared to *I_geo_*. The EF shows that 21.7% of sampling stations in BMR are classified as a very high enrichment of arsenic such as at S5, S6, S10, S11 and S12 while the rest of the stations (78.3%) are classified as extremely high enrichment. Most of the stations with extremely high enrichment classification were recorded at the southern part of BMR such as S17 (97.29), S16 (85.96), S18 (82.37) and only S1 (93.06) represents the northern part of BMR. However, based on *I_geo_* assessment, S6, S10, S11 and S12 were considered unpolluted and S5 is considered as unpolluted to moderately polluted. In general, *I_geo_* index revealed that 34.8% of stations in BMR were considered unpolluted, 52.2% as unpolluted to moderately polluted and 13.0% as moderately polluted.

## DISCUSSION

High concentration of arsenic in the southern part of BMR particularly at S16 (10.88 ± 1.81 mg kg^−1^), S18 (9.22 ± 1.70 mg kg^−1^), S14 (6.47 ± 1.76 mg kg^−1^), S13 (6.26 ± 1.8 mg kg^−1^) and S17 (5.66 ± 1.25 mg kg^−1^) can be explained due to the presence of anthropogenic activities in the area. Nevertheless, the level of arsenic in BMR is lower compared to Lake Bera, Pahang (59.89 ± 4.00 mg kg^−1^) (Gharibreza *et al*. 2013). Other lakes also recorded a higher value of arsenic such as lake Taihu, China (11.1 mg kg^−1^) (Qin *et al*. 2016) and Lake Bosten, China (16.99–89.16 mg kg^−1^) (Liu *et al*. 2015). The agricultural sector that is surrounding the lake basin constitutes a vast area such as rubber plantation (98.9 km^2^), palm oil (48.3 km^2^) and paddy field (15.5 km^2^) (Hidzrami 2010). This is believed to be one of the main factors that contribute to the enrichment of arsenic in BMR.

Trace elements are known to accumulate in the sediment through chemical and physical binding or by adsorption onto organic and inorganic particles due to these anthropogenic activities (Rieuwerts 2015; Rieuwerts *et al*. 1998; Sakan *et al*. 2012). Fertilizers, pesticides and animal feeding which contain arsenic are widely used in the agriculture sector and can be carried into the lake through surface runoff during precipitation. Some of the fertilizer might contain monosodium and disodium methylarsonate which can influence the pH of sediment and eventually affect the retention, mobility and bioavailability of arsenic (Gorny *et al*. 2015; Hooda 2010; Kadhum *et al*. 2015). pH is regarded as one of the prominent factors that governed the behaviour of arsenic in sediment. For an element that exists as anion such as arsenic, a decrease in pH will increase the sorption process because soil colloids are increasingly acquired an additional positive charge (Hooda 2010). In this part of the lake, situated a four-star lakefront resort and luxury private residential houses. Thus, there is a possibility that the greywater from these places being released into the lake and act as a nonpoint source of pollutant-containing arsenic.

Meanwhile, a high concentration of arsenic in S1 is attributed to the presence of angler jetty where residue from boats and oil spills might contain arsenic and ultimately accumulate in the sediment (Tornero & Hanke 2016). This is shown by the high value of EF (93.06) and *I_geo_* (1.02). It is important to note that S1 also contains high organic matter (39.37%). The same condition could also be observed in the southern part of the lake where most stations with high arsenic concentration similarly recorded a higher organic matter as represented by S18 (46.15%), S16 (43.11%), S17 (38.35%) and S15 (30.88%). This is proven by the Spearman rank analysis which shows a positive correlation between the level of organic matter with the concentration of arsenic. The organic matter composed of three main substances: (i) living organism, (ii) soluble biochemicals (i.e., carbohydrates, organic acids, protein, amino acids and polysaccharides), and (iii) insoluble humic materials. Sorption of the trace elements can occur through acid functional groups like amino groups, carboxylic, phenolic and alcoholic which originated from the biochemical and humic substances in the organic matter (Bauer & Blodau 2006; Draszawka-Bolzan 2015; Gorny *et al*. 2015). Organic matter can alter the solubility of potentially toxic elements, altering the distribution between the oxidised and reduced form of these elements (Hooda 2010; Rieuwerts *et al*. 1998; Shtangeeva 2005). Organic matters are redox reactive which can facilitate the release and redox transformation of less mobile solid-phase As(V) into As(III) which is more soluble and mobile. As(III) then can diffuse upward to be released into the water collum or re-precipitate in the oxic environment (Galloway *et al*. 2018). Thus, leads to a substantial increase of arsenic enrichment in the sediment surface.

Another factor that might contribute to the high level of arsenic in BMR is the presence of high concentration of ferum. This study indicates that the soil in BMR contains a high concentration of ferum. A previous study on the level of Fe in water collum in BMR also revealed that Fe concentration is beyond the permissible limit due to the type of soil in this area, which is primarily composed of laterite that are known to be rich in ferum (Shuhaimi-Othman *et al*. 2010). Fe(III) oxides are known to have a high affinity for As species because both As(III) and As(V) are strongly chemisorbed especially with decreasing pH (Hooda 2010; Wang & Mulligan 2006).

The high disparity between the value *I_geo_* and EF was predictable as many authors also reported the high disparity of EF and *I_geo_* (Haris & Aris 2013; Loska *et al*. 2003; Nowrouzi & Pourkhabbaz 2014; Shafie *et al*. 2013; Sukri *et al*. 2018). This is due to the choice of reference element during the calculation of EF. Therefore, usage of average crust concentration may lead to over or underestimation of EF. The EF values show that as the values of metals vary the classification of contamination levels vary. However, this is not the case when dealing with the *I_geo_* where the classification of contamination does not always vary as the contents of metal vary. Thus usage of *I_geo_* is more consistent and preferable (Ghrefat *et al*. 2016; Ghrefat *et al*. 2011). Nevertheless, this index is still important in providing an easy assessment of sediment quality.

## CONCLUSION

As the oldest man-made reservoir in Peninsular Malaysia, BMR can be used as an example of the long term impact of anthropogenic activities on the arsenic contamination on the surface sediment of other freshwater reservoirs in this country. The result of this study revealed that the enrichment of arsenic in surface sediment of BMR was concentrated at the south of the lake where most of the anthropogenic activities could be observed. Based on the result of this study, *I_geo_* is more appropriate and reliable to be used as a tool for classification of the enrichment level of arsenic in BMR compared to EF as EF is highly influenced by the concentration of the reference element used. By providing this information, it will help the authority to take a proactive and corrective measure to reduce the influx of pollutant-containing arsenic in the reservoir in the future.

## Figures and Tables

**Table 1:** The degree of metal enrichment based on Geo-accumulation (*I_geo_*) classification.

*I_geo_* value	Class	Designation of sediment quality
*I_geo_* ≤ 0	0	Unpolluted
0 ≤ *I_geo_* ≤ 1	1	Unpolluted to moderately polluted
1 ≤ *I_geo_* ≤ 2	2	Moderately polluted
2 ≤ *I_geo_* ≤ 3	3	Moderately to strongly polluted
3 ≤ *I_geo_* ≤ 4	4	Strongly polluted
4 ≤ *I_geo_* ≤ 5	5	Strongly to extremely polluted
*I_geo_* > 6	6	Extremely polluted

**Table 2:** The degree of metal enrichment based on the enrichment factor (EF) classification.

EF classification	Degree of enrichment
< 2	Depletion to mineral enrichment
2 ≤ EF < 5	Moderate enrichment
5 ≤ EF < 20	Significant enrichment
20 ≤ EF < 40	Very high enrichment
EF > 40	Extremely high enrichment

**Table 3:** Descriptive statistics for sediment and water in BMR.

Matrix		Mean	SD	Max	Min	CV%
Sediment	pH	4.42	0.71	7.38	3.52	16.06
	Conductivity (μS cm^−1^)	205.70	164.07	727.20	11.50	79.76
	Redox potential (mV)	121.77	42.45	173.60	–46.00	34.86
	Organic matter (%)	25.35	9.34	46.15	16.30	36.84
	Iron (Fe) mg kg^−1^	1995.10	593.82	3206.39	893.93	29.76
	Arsenic (As) mg kg^−1^	4.302	2.43	12.16	1.07	56.49

Water	pH	6.47	0.60	7.58	5.47	9.27
	Conductivity (μS cm^−1^)	25.35	3.80	29.29	17.31	15.00
	Redox potential (mV)	–62.53	34.61	59.77	–62.53	55.35
	Dissolved oxygen (mg L^−1^)	6.85	0.72	8.05	5.27	10.51

## References

[b1-tlsr-31-3-109] Adebola BAK, Joseph KS, Adebayo AO (2018). Integrated assessment of the heavy metal pollution status and potential ecological risk in the Lagos Lagoon, South West, Nigeria. Human and Ecological Risk Assessment: An International Journal.

[b2-tlsr-31-3-109] Adel Mashaan R, Yaaroub Faleh A, Abd-Al-Husain NAO, Mustafa N (2011). Using pollution load index (PLI) and geoaccumulation index ( I-Geo ) for the assessment of heavy metals pollution in Tigris River sediment in Baghdad region. Journal of Al-Nahrain University.

[b3-tlsr-31-3-109] Akinbile CO, Yusoff MS, Talib SHA, Hasan ZA, Ismail WR, Sansudin U (2013). Qualitative analysis and classification of surface water in Bukit Merah Reservoir in Malaysia. Water Science and Technology: Water Supply.

[b4-tlsr-31-3-109] Alam MGM, Allinson G, Stagnitti F, Tanaka A, Westbrooke M (2002). Arsenic contamination in Bangladesh groundwater: A major environmental and social disaster. International Journal of Environmental Health Research.

[b5-tlsr-31-3-109] Ali MM, Ali ML, Islam MS, Rahman MZ (2016). Preliminary assessment of heavy metals in water and sediment of Karnaphuli River, Bangladesh. Environmental Nanotechnology, Monitoring & Management.

[b6-tlsr-31-3-109] Alves CM, Ferreira CMH, Soares HMVM (2018). Relation between different metal pollution criteria in sediments and its contribution on assessing toxicity. Chemosphere.

[b7-tlsr-31-3-109] Ambak MA, Jalal KCA (2006). Sustainability issues of reservoir fisheries in Malaysia. Aquatic Ecosystem Health and Management.

[b8-tlsr-31-3-109] Bauer M, Blodau C (2006). Mobilization of arsenic by dissolved organic matter from iron oxides, soils and sediments. Science of the Total Environment.

[b9-tlsr-31-3-109] Bosch AC, O’Neill B, OSigge G, Kerwathb SE, Hoffman LC (2016). Heavy metals in marine fish meat and consumer health: A review. Journal of the Science of Food and Agriculture.

[b10-tlsr-31-3-109] Cagnin RC, Quaresma VS, Chaillou G, Franco T, Bastos AC (2017). Arsenic enrichment in sediment on the eastern continental shelf of Brazil. Science of the Total Environment.

[b11-tlsr-31-3-109] Draszawka-Bolzan B (2015). Heavy metals in soils. World News of Natural Sciences.

[b12-tlsr-31-3-109] Galloway JM, Swindles GT, Jamieson HE, Palmer M, Parsons MB, Sanei H, Macumber AL (2018). Organic matter control on the distribution of arsenic in lake sediments impacted by ~ 65 years of gold ore processing in subarctic Canada. Science of the Total Environment.

[b13-tlsr-31-3-109] Gharibreza M, Ashraf M, Yusoff I, John KR (2013). An evaluation of Bera Lake (Malaysia) sediment contamination using sediment quality guidelines. Journal of Chemistry.

[b14-tlsr-31-3-109] Ghrefat HA, Abu-Rukah Y, Rosen MA (2011). Application of geoaccumulation index and enrichment factor for assessing metal contamination in sediments of Kafrain Dam, Jordan. Ennvironmental Monitoring Assessment.

[b15-tlsr-31-3-109] Ghrefat HA, Waheidi M, El Batayneh A, Nazzal Z, Zumlot T, Mogren S (2016). Pollution assessment of arsenic and other selected elements in the groundwater and soil of the Gulf of Aqaba, Saudi Arabia. Environmental Earth Sciences.

[b16-tlsr-31-3-109] Goher ME, Farhat HI, Abdo MH, Salem SG (2014). Metal pollution assessment in the surface sediment of Lake Nasser, Egypt. Egyptian Journal of Aquatic Research.

[b17-tlsr-31-3-109] Gorny J, Billon G, Lesven L, Dumoulin D, Madé B, Noiriel C (2015). Arsenic behavior in river sediments under redox gradient: A review. Science of the Total Environment.

[b18-tlsr-31-3-109] Habib J, Sadigheh J, Mohammad Ali K (2018). Assessment of heavy metal pollution and ecological risk in marine sediments (A case study: Persian Gulf). Human and Ecological Risk Assessment.

[b19-tlsr-31-3-109] Haris H, Aris AZ (2013). The geoaccumulation index and enrichment factor of mercury in mangrove sediment of Port Klang, Selangor, Malaysia. Arabian Journal of Geosciences.

[b20-tlsr-31-3-109] Hassan MM, Atkins PJ, Dunn CE, Hassan MM, Atkins PJ, Dunn CE (2011). The spatial pattern of risk from arsenic poisoning: A Bangladesh case study. Journal of Environmental Science and Health, Part A.

[b21-tlsr-31-3-109] Hatje V, Macedo S, Jesus RMD, Cotrim G, Garcia KS, De Queiroz AF, Ferreira SLC (2010). Inorganic As speciation and bioavailability in estuarine sediments of Todos os Santos Bay, BA, Brazil. Marine Pollution Bulletin.

[b22-tlsr-31-3-109] Hidzrami SA (2010). Bukit Merah Lake Brief. National seminar on managing lakes and their basin for sustainable use: Current status of selected lake in Malaysia.

[b23-tlsr-31-3-109] Hooda PS (2010). Trace elements in soils.

[b24-tlsr-31-3-109] Huang Z, Tang Y, Zhang K, Chen Y, Wang Y, Kong L, You T (2016). Environmental risk assessment of manganese and its associated heavy metals in a stream impacted by manganese mining in South China. Human and Ecological Risk Assessment.

[b25-tlsr-31-3-109] Islam MS, Hossain MB, Matin A, Sarker MSI (2018). Assessment of heavy metal pollution, distribution and source apportionment in the sediment from Feni River estuary, Bangladesh. Chemosphere.

[b26-tlsr-31-3-109] Ismail WR, Najib SAM (2011). Sediment and nutrient balance of Bukit Merah Reservoir, Perak (Malaysia). Lake & Reservoirs: Research and Management.

[b27-tlsr-31-3-109] Jahan S, Strezov V (2018). Comparison of pollution indices for the assessment of heavy metals in the sediments of seaports of NSW, Australia. Marine Pollution Bulletin.

[b28-tlsr-31-3-109] Kadhum SA, Ishak MY, Zulkifli SZ, Hashim R (2015). Evaluation of the status and distributions of heavy metal pollution in surface sediments of the Langat River Basin in Selangor Malaysia. Marine Pollution Bulletin.

[b29-tlsr-31-3-109] Kapaj S, Peterson H, Liber K, Bhattacharya P (2006). Human health effects from chronic arsenic poisoning: A review. Journal of Environmental Science and Health - Part A Toxic/Hazardous Substances and Environmental Engineering.

[b30-tlsr-31-3-109] Khodami S, Surif M, Wan Maznah WO, Daryanabard R (2017). Assessment of heavy metal pollution in surface sediments of the Bayan Lepas area, Penang, Malaysia. Marine Pollution Bulletin.

[b31-tlsr-31-3-109] Liu Y, Mu S, Bao A, Zhang D, Pan X (2015). Effects of salinity and (an)ions on arsenic behavior in sediment of Bosten Lake, Northwest China. Environmental Earth Sciences.

[b32-tlsr-31-3-109] Loska K, Wiechuła D, Barska B, Cebula E, Chojnecka A (2003). Assessment of arsenic enrichment of cultivated soils in Southern Poland. Polish Journal of Environmental Studies.

[b33-tlsr-31-3-109] Mudhoo A, Sharma SK, Garg VK (2011). Arsenic: An overview of applications, health, and environmental concerns and removal processes. Critical Review in Environmental Science and Technology.

[b34-tlsr-31-3-109] Muller G (1969). Index of geoaccumulation in sediments of the Rhine River. Geojournal.

[b35-tlsr-31-3-109] Nowrouzi M, Pourkhabbaz A (2014). Application of geoaccumulation index and enrichment factor for assessing metal contamination in the sediments of Hara Biosphere Reserve, Iran. Chemical Speciation and Bioavailability.

[b36-tlsr-31-3-109] Qin S, Shiming D, Yan W, Lv X, Dan W, Jing C, Chaosheng Z (2016). In-situ characterization and assessment of arsenic mobility in lake. Environmental Pollution.

[b37-tlsr-31-3-109] Radojevic M, Bashkin VN (2006). Practical environmental analysis.

[b38-tlsr-31-3-109] Rajeshkumar S, Liu Y, Zhang X, Ravikumar B, Bai G, Li X (2018). Studies on seasonal pollution of heavy metals in water, sediment, fish and oyster from the Meiliang Bay of Taihu Lake in China. Chemosphere.

[b39-tlsr-31-3-109] Rasheed H, Slack R, Kay P (2016). Human health risk assessment for arsenic: A critical review. Critical Reviews in Environmental Science and Technology.

[b40-tlsr-31-3-109] Uddin Riaz, Naz Hasan H (2011). Arsenic poisoning in Bangladesh. Oman Medical Journal.

[b41-tlsr-31-3-109] Rieuwerts JS (2015). The mobility and bioavailability of trace metals in tropical soils: A review. Chemical Speciation and Bioavailability.

[b42-tlsr-31-3-109] Rieuwerts JS, Thornton I, Farago ME, Ashmore MR (1998). Factors influencing metal bioavailability in soils: Preliminary investigations for the development of a critical loads approach for metals. Chemical Speciation and Bioavailability.

[b43-tlsr-31-3-109] Roy SP (2010). Overview of heavy metals and aquatic environment with notes on their recovery. Ecoscan: An International Quarterly Journal of Environmental Sciences.

[b44-tlsr-31-3-109] Sakan SM, ĐorĐević DS, Lazić MM, Tadić MM (2012). Assessment of arsenic and mercury contamination in the Tisa River sediments and industrial canal sediments (Danube alluvial formation), Serbia. Journal of Environmental Science and Health - Part A Toxic/Hazardous Substances and Environmental Engineering.

[b45-tlsr-31-3-109] Sanyal T, Kaviraj A, Saha S (2017). Toxicity and bioaccumulation of chromium in some freshwater fish. Human and Ecological Risk Assessment.

[b46-tlsr-31-3-109] Shafie NA, Aris AZ, Zakaria MP, Haris H, Wan YL, Isa NM (2013). Application of geoaccumulation index and enrichment factors on the assessment of heavy metal pollution in the sediments. Journal of Environmental Science and Health - Part A Toxic/Hazardous Substances and Environmental Engineering.

[b47-tlsr-31-3-109] Shtangeeva I (2005). Trace and ultratrace elements in plant and soil.

[b48-tlsr-31-3-109] Shuhaimi-Othman M, Ahmad AK, Norziana G (2010). Kepekatan logam di Tasik Bukit Merah, Perak. Sains Malaysiana.

[b49-tlsr-31-3-109] Siti Hidayah AT, Yusoff MS, Zorkeflee AH (2012). Modeling of sedimentation pattern in Bukit Merah Reservoir, Perak, Malaysia. Procedia Engineering.

[b50-tlsr-31-3-109] Sukri NS, Aspin SA, Kamarulzaman NL, Jaafar NF, Rozidaini MG, Shafiee NS, Siti Hajar Y (2018). Assessment of metal pollution using enrichment factor (EF) and pollution load index (PLI) in sediments of selected terengganu, Malaysia. Malaysian Journal of Fundamental and Applied Sciences.

[b51-tlsr-31-3-109] Tornero V, Hanke G (2016). Chemical contaminants entering the marine environment from sea-based sources: A review with a focus on European seas. Marine Pollution Bulletin.

[b52-tlsr-31-3-109] Varol M (2011). Assessment of heavy metal contamination in sediments of the Tigris River (Turkey) using pollution indices and multivariate statistical techniques. Journal of Hazardous Materials.

[b53-tlsr-31-3-109] Wang H, Liu R, Wang Q, Xu F, Men C, Shen Z (2016). Bioavailability and risk assessment of arsenic in surface sediments of the Yangtze River estuary. Marine Pollution Bulletin.

[b54-tlsr-31-3-109] Wang S, Mulligan CN (2006). Effect of natural organic matter on arsenic mobilization from mine tailings. Environmental Geochemistry and Health.

[b55-tlsr-31-3-109] Yang H, Lee C, Chiang Y, Jean J, Shau Y, Takazawa E, Jiang W (2016). Distribution and hosts of arsenic in a sediment core from the Chianan Plain in SW Taiwan: Implications on arsenic primary source and release mechanisms. Science of the Total Environment.

[b56-tlsr-31-3-109] Zahra A, Hashmi MZ, Malik RN, Ahmed Z (2014). Enrichment and geo-accumulation of heavy metals and risk assessment of sediments of the Kurang Nallah-Feeding tributary of the Rawal Lake Reservoir, Pakistan. Science of the Total Environment.

